# Adherence to a modified Mediterranean diet and in association with asthma and wheezing in schoolchildren: A cross-sectional study^☆^

**DOI:** 10.1016/j.waojou.2024.100948

**Published:** 2024-10-18

**Authors:** Faezeh Poursoleiman, Bahareh Sasanfar, Nasrin Behniafard, Zahra Nafei, Elahe Akbarian, Abbas Khalili, Amin Salehi-Abargouei

**Affiliations:** a Department of Clinical Nutrition and Dietetics, Student Research Committee, Faculty of Nutrition and Food Technology, Shahid Beheshti University of Medical Sciences, Tehran, Iran; b Research Center for Food Hygiene and Safety, School of Public Health, Shahid Sadoughi University of Medical Sciences, Yazd, Iran; c Yazd Cardiovascular Research Center, Non-communicable Diseases Research Institute, Shahid Sadoughi University of Medical Sciences, Yazd, Iran; d Department of Nutrition, School of Public Health, Shahid Sadoughi University of Medical Sciences, Yazd, Iran; e Children Growth Disorder Research Center, Shahid Sadoughi University of Medical Sciences, Yazd, Iran; f Department of Allergy and Clinical Immunology, Shahid Sadoughi University of Medical Sciences, Yazd, Iran; g Student Research Committee, Shahid Sadoughi University of Medical Sciences, Yazd, Iran; h Department of Pediatrics, Shahid Sadoughi Hospital, Shahid Sadoughi University of Medical Sciences, Yazd, Iran

**Keywords:** Asthma, Dietary pattern, Wheezing, Schoolchildren

## Abstract

**Background:**

Limited investigations have focused on the association between the Mediterranean dietary (MeD) and asthma among children and adolescents. We aimed to study the associations between a modified Mediterranean dietary pattern and asthma symptoms in children living in Iran.

**Method:**

This cross-sectional study was conducted among 7667 children and adolescence. Data on dietary intakes, asthma symptoms and other possible confounders, were collected using a questionnaire completed by parents. The relationship between Mediterranean diet (MeD) and asthma was assessed using logistic regression.

**Results:**

We found that participants in the highest quartile of MeD score had 32% lower odds of wheezing in the past 12 months in the whole population when compared with those in the lowest quartile (OR: 0.68; 95% CI: 0.51–0.90; P_trend_ < 0.001). Regarding the wheezing, a linear reducing trend was observed in girls (OR: 0.88; 95% CI: 0.62–1.25; P_trend_ = 0.04); and a significant protective association was seen among boys (OR: 0.45; 95% CI: 0.28–0.73; P_trend_ < 0.001). Analyses by gender showed, girls and boys in the highest quartile had 68% and 51% lower odds of current asthma in comparison with the first quartile.

**Conclusion:**

Mediterranean-style diet emphasizing high in fruits, vegetables, and cereals and low in fast foods is associated with reduced wheezing as an asthma-related symptom. On the other hand, meat intake has adverse correlation with asthma prevalence. No association was found between this dietary pattern and asthma. Further prospective investigations should be conducted to confirm these findings.

## Introduction

Asthma, a chronic non-communicable condition, has caused a substantial burden all around the world.^1^^,^^2^ According to the Global Burden of Disease study (GBD), in 2016 more than 300 million people lived with asthma worldwide which might increase to 400 million in 2025.^3^^,^^4^ Asthma has a wide range of prevalence in different countries from 0.2% in China to 21.5% in Australia.^5^ A higher prevalence has been seen in developed countries.^6^ Besides, asthma is one of the most growing health problems in childhood; as about 10% of children suffer from this airway inflammatory disease, globally.^7^ In Iran, the prevalence of asthma 6% and 8% among children and adolescents, respectively.^8^

Alongside with genetic susceptibility, the significant variation in prevalence and severity rate of asthma and its symptoms in geographically dispersed countries prove impressive effects of environmental factors and lifestyle on its epidemiology;^9^ hence, as a lifestyle factor, it is not far-fetched to connect dietary pattern and food intake to asthma.

In line with the increase of asthma in westernized countries over the past 4 decades,^10^ recent studies showed that caloric dense foods, processed foods, red meats, and sweets, the key components of western diet, are important diet-related risk factors in asthma prevalence and its symptoms severity both in children^11^ and adults;^12^ however, dietary patterns with higher amounts of fruits and vegetables,^13^ oily fish,^14^ olive oil,^15^ and whole meal cereals^16^ might have protective effects.^7^^,^^11^^,^^17^^,^^18^ However, there are contradictory results too.^19^^,^^20^ The Mediterranean diet (MeD) pattern, traditionally emulated by those living in Mediterranean countries such as Spain, Greece, and southern Italy, which is rich in fruits, vegetables, cereals, nuts, seeds, and olive oil and has low content of sweets, fats, and red or processed meats.^21^ Inflammatory markers like leptin, plasminogen activator inhibitor 1(PA-1), and C-reactive protein were inversely associated with the MeD.^7^ The MeD can trigger anti-inflammatory pathways^22^ and thus modulate inflammatory mediators in chronic inflammatory diseases like metabolic syndrome,^23^, ^24^, ^25^ diabetes,^26^^,^^27^ heart disease,^28^^,^^29^ and cancer,^30^^,^^31^ as well as autoimmune diseases like systemic lupus erythematosus,^32^ multiple sclerosis,^33^ and rheumatoid arthritis.^34^ A limited number of studies have tried to examine the association between adherence to the MeD and asthma, allergic rhinitis, or atopic eczema. Previous studies showed that asthma and wheezing-as a key symptom of asthma-might be mitigated by adherence to the MeD in adults and children,^22^^,^^35^ although the results were inconclusive.^10^^,^^36^ Fewer number of studies have focused on the association between a MeD diet and childhood respiratory diseases.^37^ Small sample size,^37^ different MeD scores in analyses and heterogeneity in participants might decrease the power of previous investigations.^38^ As limited investigations have focused on the association between the MeD and asthma among children and adolescents, particularly those living in the Middle East, and Iran, we aimed to study the associations between a modified Mediterranean dietary pattern and asthma symptoms in children living in Iran.

## Methods

### Study population

This cross-sectional study is a part of a cross-sectional, multi-center, epidemiological research conducted in early 2020 in Yazd, Iran (the Global Asthma Network [GAN] study). Schoolchildren aged 6–7 (as children) and 13–14 years (as adolescents), were randomly selected from 48 elementary and 36 guidance schools, respectively, using a cluster sampling design. The methodology of GAN study was according to the International Study of Asthma and Allergies in Childhood (ISAAC).^39^

### Questionnaires

Prior to the COVID-19 pandemic, parents of 6–7-year-old children completed GAN questionnaires in person at schools, which included demographic data, information on asthma and allergic symptoms, as well as its associated risk factors (n = 1046). Following school closures during the pandemic, parents of the remaining 6–7-year-olds (n = 1979) and all 13–14-year-olds (n = 7214) were invited to complete electronic questionnaires online. In total, parents of 5141 out of 7214 adolescents and 2526 out of 3025 children successfully completed the questionnaires. The response rate among adolescents was 71.5%, while among children, it was 83.5%. In order to approve the GAN questionnaire, the original version was translated into Persian and then returned to English and sent to the GAN principal investigators for validity checking. The reliability of the translated version was confirmed by a study conducted on 100 selected subjects using Cronbach's alpha. The alpha coefficient for asthma symptoms were estimated to be 0.862, which showed an appropriate internal consistency.

### Dietary assessment and developing a Mediterranean dietary pattern score

Dietary intakes were assessed using a multiple-choice questionnaire in the GAN study.^40^ The GAN questionnaire included Food Frequency Questionnaire (FFQ) with 26 food group items which assessed previous past twelve-month food items consumption. The Mediterranean diet score was calculated for study participants using a scoring system introduced by Trichopoulou et al:^41^ fruits, vegetables, potatoes, pulses, cereals, pasta, rice, and fish were considered “pro-Mediterranean” foods and rated 0, 1, or 2 points from less frequent to more frequent intake (0 points = never or occasionally; 1 point = 1or 2 times/week; 2 points ≥3 times/week). Meat, milk, and fast foods were considered “anti-Mediterranean” and rated 0, 1, or 2 points from more frequent consumption (0 points ≥3 times/week; 1 point = 1or 2 times/week; 2 points = never or occasionally). The scores were then summed up to compute the Mediterranean diet score. The theoretical overall score for participants was ranged from 5 to 22.

### Asthma and its symptoms

Using the standard GAN questionnaire, data related to asthma symptoms, severity, and the use of asthma medications were collected. Current asthma was operationally defined as the presence of both physician-diagnosed asthma and reported wheezing or use of asthma medication within the past 12 months.

### Statistical analysis

The analyses were done in the whole population as well as stratified by sex. Individuals were categorized based on quartiles of the Mediterranean diet scores. We used independent samples *t*-test and chi-square tests to compare continuous and categorical variables in those with and without asthma, respectively. Multivariable logistic regression models were used to assess the association between adherence to the Mediterranean diet scores and odds of asthma confirmed by a physician, current asthma, and wheezing in the past 12 months. The analyses was adjusted for age and sex in the first model, in addition to watching television and computer use in the second model and in the last model body mass index (BMI) was additionally adjustment for BMI. STATA version 14 was used for all analyses (State Crop., College Station, TX). P-value < 0.05 was considered as statistically significant.

## Results

Totally 4.22% of participants had previously received a confirmed asthma diagnosis from physicians. General characteristics of study participants in cases with and without confirmed asthma are presented in Table 1. Participants with confirmed asthma were more likely to be male (58% vs. 43.9%, P < 0.001), older (11.7 vs. 10.9 years, P < 0.001), expressed wheezing in the past 12 months (17.2% vs. 7.5%, P < 0.001) and use asthma medications (17.5% vs. 1.8%, P < 0.001) than those without asthma.

**Table 1:** General characteristics of subjects according to the asthma confirmed by a physician

Variables	Asthma confirmed by a physician		*P*-value^a^
	No (n = 7343)	Yes (n = 324)	
Male	3226 (43.9)	188 (58.0)	<0.001
Age (years)	10.9 ± 3.37	11.7 ± 2.94	<0.001
BMI (kg/m^2^)	18.9 ± 10.4	19.1 ± 4.18	0.35
watch TV and computer use			
2–4 h	3945 (53.7)	163 (50.3)	0.38
5–8 h	2463 (33.5)	103 (34.8)	
9–14 h	935 (12.7)	48 (14.8)	
Wheezing b			
Yes	553 (7.5)	56 (17.2)	<0.001
No	6790 (92.4)	268 (82.7)	
Use of asthma Medications			
Yes	134 (1.8)	57 (17.5)	<0.001
No	7209 (98.1)	267 (82.4)	

BMI, body mass index.

Values are mean ± SD or percentages.

a Chi-square Test for ordinal qualitative variables and independent samples student t-test for continuous variables.

b In the past 12 months.

The frequency of MeD components intake in those with and without confirmed asthma and wheezing are presented in Table 2. The frequency of meat consumption was significantly different in those with confirmed asthma compared to those without (P < 0.05). The frequency of fruits, vegetables, cereals, and fast foods intake was different between those with and without wheezing in the past 12 months (P < 0.05, Table 2).

**Table 2:** Frequency of dietary intake of schoolchildren 6–7 and 13–14 ages, according to confirmed asthma and wheezing condition^a^

Variables	Asthma confirmed by a physician		*P*-value^b^	wheezing		*P*-value^b^
	No (n = 7343)	Yes (n = 324)		No (n = 7058)	Yes (n = 609)	
Fruits			0.58			0.004
Never	256 (3.4)	12 (3.7)		239 (3.39)	29 (4.7)	
Weekly	1248 (17.0)	62 (19.1)		1182 (16.7)	128 (21.0)	
Daily	5839 (79.5)	250 (77.2)		5637 (79.8)	452 (74.2)	
Vegetables			0.30			<0.001
Never	1852 (25.2)	94 (29.0)		1750 (24.7)	196 (32.1)	
Weekly	4049 (55.1)	171 (52.7)		3905 (55.3)	315 (51.7)	
Every day	1442 (19.6)	59 (18.2)		1403 (19.8)	98 (16.0)	
Potatoes			0.77			0.08
Never	412 (5.6)	19 (5.8)		390 (5.5)	41 (6.7)	
Weekly	4721 (64.2)	202 (62.3)		4517 (64.0)	406 (66.6)	
Every day	2210 (30.1)	103 (31.7)		2151 (30.4)	162 (26.6)	
Legumes			0.69			0.17
Never	276 (3.7)	10 (3.0)		257 (3.6)	29 (4.7)	
Weekly	4271 (58.1)	195 (60.1)		4101 (58.1)	365 (59.9)	
Every day	2796 (38.0)	119 (36.7)		2700 (38.2)	215 (35.3)	
Cereals			0.23			0.002
Never	2401 (32.7)	114 (35.1)		2278 (32.2)	237 (38.9)	
Weekly	3713 (50.5)	167 (51.5)		3590 (50.8)	290 (47.6)	
Every day	1229 (16.7)	43 (13.2)		1190 (16.8)	82 (13.4)	
Pasta			0.96			0.69
Never	1848 (25.1)	81 (25.0)		1767 (25.0)	162 (26.6)	
Weekly	5108 (69.5)	227 (70.0)		4920 (69.7)	415 (68.1)	
Every day	387 (5.2)	16 (4.9)		371 (5.2)	32 (5.2)	
Rice			0.33			0.50
Never	123 (1.6)	2 (0.62)		115 (1.6)	10 (1.6)	
Weekly	956 (13.0)	43 (13.2)		929 (13.1)	70 (11.4)	
Every day	6264 (85.3)	279 (86.1)		6014 (85.2)	529 (86.8)	
Fish			0.28			0.07
Never	4291 (58.4)	202 (62.3)		4110 (58.2)	383 (62.8)	
Weekly	2934 (39.9)	119 (36.7)		2834 (40.1)	219 (35.9)	
Every day	118 (1.6)	3 (0.93)		114 (1.6)	7 (1.1)	
Milk			0.96			0.61
Never	1276 (17.3)	57 (17.5)		1219 (17.2)	114 (18.7)	
Weekly	3861 (52.5)	168 (51.8)		3718 (52.8)	311 (51.0)	
Every day	2206 (30.0)	99 (30.5)		2121 (30.0)	184 (30.2)	
Meats			0.03			0.59
Never	519 (7.0)	21 (6.4)		496 (7.0)	44 (7.2)	
Weekly	3546 (48.2)	180 (55.5)		3442 (48.7)	284 (46.6)	
Every day	3278 (44.6)	123 (37.9)		3120 (44.2)	281 (46.1)	
Fast foods			0.53			0.01
Never	4120 (56.1)	188 (58.0)		4001 (56.6)	307 (50.4)	
Weekly	2961 (40.3)	122 (37.6)		2806 (39.7)	277 (45.4)	
Every day	262 (3.5)	14 (4.3)		251 (3.5)	25 (4.1)	
Olive oil			0.55			0.24
Never	5055 (68.8)	219 (67.5)		4838 (68.5)	436 (71.5)	
Weekly	1583 (21.5)	68 (20.9)		1528 (21.6)	123 (20.2)	
Every day	705 (9.6)	37 (11.4)		692 (9.8)	50 (8.2)	

a All analyses were done using Chi-square test.

b *P*-value <0.05 was considered as statistically significant.

Crude and multivariable-adjusted ORs and 95% CIs for the association between adherence to the MeD and odds of wheezing in the past 12 months are presented in Table 3. After adjustment for all possible confounders, we found that those in the highest quartile of MeD score had 32% lower odds of wheezing in the past 12 months in the whole population when compared with those in the lowest quartile (OR: 0.68; 95% CI: 0.51–0.90; P_trend_ < 0.001). When the analyses were done separately by gender, although those in the highest quartile were not significantly different regarding the odds of wheezing, a linear reducing trend was observed in girls (OR: 0.88; 95% CI: 0.62–1.25; P_trend_ = 0.04); and a significant protective association was seen among boys (OR: 0.45; 95% CI: 0.28–0.73; P_trend_ < 0.001).

**Table 3:** Odds ratios and 95% CIs for wheezing in the past 12 months across quartiles of Mediterranean diet scores in the total population as well as based on gender

	Quartile of Mediterranean diet score				*P*_trend_
	Q1	Q2	Q3	Q4	
	OR (95% CI)	OR (95% CI)	OR (95% CI)	OR (95% CI)	
Total participants (n with/without wheezing)	264/2584	127/1296	149/2234	69/944	
Crude	1.00	0.95 (0.76–1.19)	0.65 (0.52–0.80)^d^	0.71 (0.54–0.94)^d^	<0.001
Model 1^a^	1.00	0.95 (0.76–1.18)	0.64 (0.52–0.79)^d^	0.71 (0.54–0.94)^d^	<0.001
Model 2^b^	1.00	0.96 (0.76–1.20)	0.63 (0.51–0.78)^d^	0.68 (0.51–0.90)^d^	<0.001
Model 3^c^	1.00	0.96 (0.76–1.20)	0.63 (0.51–0.78)^d^	0.68 (0.51–0.90)^d^	<0.001
Girls (n with/without wheezing)	130/1417	67/720	74/1245	47/553	
Crude	1.00	1.01 (0.74–1.38)	0.64 (0.48–0.87)^d^	0.92 (0.65–1.31)	0.06
Model 1	1.00	1.00 (0.73–1.36)	0.63 (0.47–0.85)^d^	0.91 (0.64–1.30)	0.05
Model 2	1.00	1.02 (0.75–1.40)	0.64 (0.47–0.86)^d^	0.88 (0.62–1.25)	0.04
Model 3	1.00	1.02 (0.75–1.39)	0.64 (0.47–0.86)^d^	0.88 (0.62–1.25)	0.04
Boys (n with/without wheezing)	134/1167	60/576	75/989	22/391	
Crude	1.00	0.90 (0.65–1.24)	0.66 (0.49–0.88)^d^	0.49 (0.30–0.78)^d^	<0.001
Model 1	1.00	0.90 (0.65–1.24)	0.64 (0.48–0.87)^d^	0.49 (0.31–0.79)^d^	<0.001
Model 2	1.00	0.89 (0.65–1.24)	0.63 (0.46–0.85)^d^	0.45 (0.28–0.73)^d^	<0.001
Model 3	1.00	0.89 (0.65–1.24)	0.63 (0.47–0.85)^d^	0.45 (0.28–0.73)^d^	<0.001

a Model 1: adjusted for age and sex (for total participants).

b Model 2: further adjusted for watching TV & computer use.

c Model 3: additionally, adjusted for BMI.

d Confidence interval was considered as statistically significant.

Crude and multivariable-adjusted ORs and 95% CIs for the association between adherence to the MeD and odds of current asthma are presented in Table 4. Neither in crude nor in adjusted models, had we found significant association between adherence to the MeD and odds of current asthma in the whole population. When the analyses were done separately by gender, girls and boys in the highest quartile had 68% and 51% lower odds of current asthma in comparison with the first quartile, however no significant association was found [for girls: (OR: 0.32; 95% CI: 0.04–2.64) and for boys: (OR: 0.49; 95% CI: 0.14–1.70)] in the fully adjustment model.

**Table 4:** Odds ratios and 95% CIs for current asthma across quartiles of Mediterranean diet scores in the total population as well as based on gender

	Quartile of Mediterranean diet score				*P*_trend_
	Q1	Q2	Q3	Q4	
	OR (95% CI)	OR (95% CI)	OR (95% CI)	OR (95% CI)	
Total participants (n with/without current asthma)	25/2478	13/1251	14/2153	4/908	
Crude	1.00	1.03 (0.52–2.02)	0.64 (0.33–1.24)	0.43 (0.15–1.25)	0.06
Model 1^a^	1.00	1.04 (0.53–2.05)	0.66 (0.34–1.28)	0.44 (0.15–1.27)	0.07
Model 2^b^	1.00	1.04 (0.53–2.05)	0.66 (0.34–1.28)	0.44 (0.15–1.27)	0.07
Model 3^c^	1.00	1.04 (0.53–2.05)	0.66 (0.34–1.28)	0.44 (0.15–1.27)	0.07
Girls (n with/without current asthma)	8/1357	7/694	5/1199	1/532	
Crude	1.00	1.71 (0.61–4.73)	0.70 (0.23–2.16)	0.31 (0.03–2.55)	0.23
Model 1	1.00	1.75 (0.63–4.85)	0.73 (0.23–2.25)	0.32 (0.04–2.58)	0.25
Model 2	1.00	1.72 (0.62–4.79)	0.72 (0.23–2.23)	0.33 (0.04–2.65)	0.25
Model 3	1.00	1.71 (0.61–4.74)	0.72 (0.23–2.22)	0.32 (0.04–2.64)	0.25
Boys (n with/without current asthma)	17/1121	6/557	9/954	3/376	
Crude	1.00	0.71 (0.27–1.81)	0.62 (0.27–1.40)	0.52 (0.15–1.80)	0.17
Model 1	1.00	0.71 (0.27–1.81)	0.63 (0.28–1.43)	0.50 (0.14–1.74)	0.16
Model 2	1.00	0.71 (0.27–1.81)	0.63 (0.27–1.42)	0.49 (0.14–1.70)	0.15
Model 3	1.00	0.71 (0.27–1.81)	0.62 (0.27–1.42)	0.49 (0.14–1.70)	0.15

a Model 1: adjusted for age and sex (for total participants).

b Model 2: further adjusted for watching TV & computer use.

c Model 3: additionally, adjusted for BMI.

Crude and multivariable-adjusted ORs and 95% CIs for the association between adherence to the MeD and odds of asthma confirmed by a doctor are presented in Table 5. After controlling for the potential confounders, we found no significant association between adherence to the MeD and odds of asthma in the whole population (OR: 0.85; 95% CI: 0.59–1.22). There was also no significant association between adherence to the MeD and odds of asthma among either gender (for girls: [OR: 0.81; 95% CI: 0.50–1.33] and for boys: [OR: 0.87; 95% CI: 0.51–1.50]).

**Table 5:** Odds ratios and 95% CIs for asthma confirmed by a physician across quartiles of Mediterranean diet scores in the total population as well as based on gender

	Quartile of Mediterranean diet score				*P*_trend_
	Q1	Q2	Q3	Q4	
	OR (95% CI)	OR (95% CI)	OR (95% CI)	OR (95% CI)	
Total participants (n with/without confirmed asthma)	131/2717	58/1365	95/2288	40/973	
Crude	1.00	0.88 (0.64–1.20)	0.86 (0.65–1.12)	0.85 (0.59–1.22)	0.24
Model 1^a^	1.00	0.87 (0.63–1.19)	0.84 (0.64–1.10)	0.85 (0.59–1.22)	0.22
Model 2^b^	1.00	0.87 (0.63–1.19)	0.84 (0.64–1.10)	0.85 (0.59–1.22)	0.21
Model 3^c^	1.00	0.87 (0.63–1.19)	0.84 (0.64–1.10)	0.85 (0.59–1.22)	0.21
Girls (n with/without confirmed asthma)	68/1479	33/754	51/1268	22/578	
Crude	1.00	0.95 (0.62–1.45)	0.87 (0.60–1.26)	0.82 (0.50–1.35)	0.36
Model 1	1.00	0.92 (0.60–1.40)	0.84 (0.58–1.22)	0.81 (0.49–1.32)	0.29
Model 2	1.00	0.91 (0.59–1.40)	0.84 (0.57–1.21)	0.81 (0.49–1.33)	0.29
Model 3	1.00	0.92 (0.60–1.41)	0.84 (0.58–1.22)	0.81 (0.50–1.33)	0.30
Boys (n with/without confirmed asthma)	63/1238	25/611	44.1020	18/395	
Crude	1.00	0.80 (0.50–1.29)	0.84 (0.57–1.25)	0.89 (0.52–1.53)	0.48
Model 1	1.00	0.80 (0.50–1.28)	0.84 (0.56–1.24)	0.90 (0.52–1.54)	0.49
Model 2	1.00	0.80 (0.49–1.28)	0.83 (0.56–1.24)	0.88 (0.51–1.50)	0.44
Model 3	1.00	0.80 (0.49–1.28)	0.83 (0.56–1.23)	0.87 (0.51–1.50)	0.43

a Model 1: adjusted for age and sex (for total participants).

b Model 2: further adjusted for watching TV & computer use.

c Model 3: additionally, adjusted for BMI.

## Discussion

In the current cross-sectional study, we explored the association between adherence to Mediterranean diet (MeD) and its specified food items with current asthma, confirmed asthma, and wheezing in Iranian school children. Our findings indicate that higher adherence to the MeD was inversely associated with wheezing in the past 12 months, irrespective of gender, and this trend was consistent when analyzed separately for girls and boys. However, we did not find significant associations between MeD adherence and current asthma or confirmed asthma.

Our results align with some previous studies that also reported no significant association between MeD score and asthma prevalence.^10^^,^^42^ Conversely, Castro-Rodriguez et al suggested a potential protective effect of the MeD against current wheezing in preschool children,^43^ while Chatzi et al found no beneficial effects for wheezing.^37^ Other studies have shown an inverse association between MeD adherence and both asthma and wheezing.^44^ For example, Arvaniti et al. using KIDMED scoring method, revealed that greater adherence to the MeD was inversely associated with ever had wheezing, exercise wheeze, ever had diagnosed asthma and with any asthma symptoms among 10–12 years old children.^45^ In an almost identical design with the current study, a significant protective effect of MeD for current asthma was shown, but no data was reported for wheezing.^20^

It was shown that anti-inflammatory diet like the MeD, characterized by high intake of fruits, vegetables, cereals, legumes, and lower intake of meats^46^ are associated with lower risk of asthma and wheezing,^47^ while westernized style diets rich in saturated fats, refined grains, red meats, and processed foods and lower intake of fibers increases risk of asthma and wheezing.^48^^,^^49^ Although recent studies have revealed that the MeD as a protection factor for the asthma prevalence and asthma outcomes, results are inconsistent. Our results showed no protective association for MeD on confirmed asthma and current asthma; however, a significant association was seen for wheezing. The fact that we have not come to the same conclusion as some articles on current asthma is probably due to different definitions. Nonhomogeneous factors were used for asthma definition, for example in a study done by Gonzales et al,^42^ current asthma was defined as “having wheezing or whistling in the chest during the last 12 months” but in another study^20^ it defined as “previously diagnosed asthma by a physician in addition to wheezing/whistling in the chest over the last 12 months”, which both are different from our definition (mentioned in method). This inconsistency may also be due to the geographical differences of studies as asthma is affected by environment.

Analysis of dietary items showed that intake of the **fruits** (daily), **vegetables** (weekly), and **cereals** (weekly) may have protective effects on odds of wheezing; but intake of **fast foods** (weekly) and **meats** (weekly) have incremental effects on odds of wheezing and confirmed asthma, respectively.

In terms of food items in MeD, Ellwood et al in a large population of children and adolescence showed protective effects for fruits and vegetables and destructive effects of fast foods on current and severe wheeze.^17^ Nkosi et al also showed that eating fast foods three or more times in week was associated with wheeze and asthma.^50^ Hosseini et al in a systematic review found a negative association between fruit but not for vegetable intake and wheezing prevalence whereas it was vice versa for asthma prevalence. On the other hand, Concomitant consumption of fruit and vegetable intake had no significant association with odds of asthma prevalence in their study.^51^ In an overview of systematic reviews an inverse association between asthma/wheezing and dietary intake of fruits and adherence to a MeD was shown.^52^ Putting together, studies support fruits and vegetables intake and a significant association between wheezing and a modest protective effect for asthma^13^ that is in the same direction of our findings. A recent meta-analysis showed that dietary meat intake may not be a risk factor for asthma in children.^53^

Plant-based foods are high in vitamins, antioxidants, phytochemicals like carotenoids and flavonoids and fibers which all act as protector against inflammation and respiratory oxidative damage, prime processes in the clinical signs of respiratory problems like asthma and wheezing.^19^^,^^54^^,^^55^ This effect seems to be more pronounced when they are in the form of whole diet and consumed together, rather than as nutrients alone.^56^ Dietary inflammatory index (DII) that presents diet's inflammatory potential also confirmed that the consumption of balanced diet rich in anti-inflammatory nutrients and low in oxidant and pro-inflammatory nutrients was associated with current ing but not current asthma, among U.S. adults and children.^57^

From the perspective of involved mechanisms, the MeD components mediate anti-inflammatory and antioxidant defense mechanisms which modulate immune responses by inhibiting pro-inflammatory mediators like cytokines, chemokines, and eicosanoid metabolites and inhibiting oxidative stress. Dietary antioxidants might inhibit NF-κB pathway, reduce cytokines as IL-6, IL-8 and T-helper 2 (crucial in asthma pathogenesis).^51^

Limitations of our study include its cross-sectional design, which precludes establishment of causal relationships. Although we employed a valid questionnaire and conducted subgroup analyses for several variables, unadjusted confounding variables could potentially influence our results. Additionally, variations in asthma definitions and potential misclassification biases should be considered when interpreting our findings.

## Conclusion

In conclusion, while our study adds to the growing body of evidence on the potential benefits of MeD in reducing respiratory symptoms like wheezing, the lack of significant associations with current and confirmed asthma warrants further investigation. Future research employing longitudinal designs and standardized asthma definitions across diverse populations will be crucial in elucidating the true impact of dietary patterns on respiratory health.

## Abbreviations

MeD, Mediterranean diet; GBD, Global Burden of Disease study; GAN, Global Asthma Network; ISAAC, International Study of Asthma and Allergies in Childhood; FFQ, Food Frequency Questionnaire; BMI, Body mass index.
